# Tadpoles of hybridising fire-bellied toads (*B*. *bombina* and *B*. *variegata*) differ in their susceptibility to predation

**DOI:** 10.1371/journal.pone.0231804

**Published:** 2020-12-07

**Authors:** Radovan Smolinský, Vojtech Baláž, Beate Nürnberger

**Affiliations:** 1 Research Facility Studenec, Institute of Vertebrate Biology, Czech Academy of Sciences, Brno, Czech Republic; 2 Faculty of Veterinary Hygiene and Ecology, University of Veterinary and Pharmaceutical Sciences, Brno, Czech Republic; University of Maine at Farmington, UNITED STATES

## Abstract

The role of adaptive divergence in the formation of new species has been the subject of much recent debate. The most direct evidence comes from traits that can be shown to have diverged under natural selection and that now contribute to reproductive isolation. Here, we investigate differential adaptation of two fire-bellied toads (Anura, Bombinatoridae) to two types of aquatic habitat. *Bombina bombina* and *B*. *variegata* are two anciently diverged taxa that now reproduce in predator-rich ponds and ephemeral aquatic sites, respectively. Nevertheless, they hybridise extensively wherever their distribution ranges adjoin. We show in laboratory experiments that, as expected, *B*. *variegata* tadpoles are at relatively greater risk of predation from dragonfly larvae, even when they display a predator-induced phenotype. These tadpoles spent relatively more time swimming and so prompted more attacks from the visually hunting predators. We argue in the discussion that genomic regions linked to high activity in *B*. *variegata* should be barred from introgression into the *B*. *bombina* gene pool and thus contribute to gene flow barriers that keep the two taxa from merging into one.

## Introduction

Adaptation to local environments is commonplace in nature [1] and drives the evolution of novel ecotypes. Whether adaptive phenotypic divergence plays an important role in the origin of new species has been the subject of much discussion [2–7]. In this case, adaptation to different ecological conditions should entail a marked fitness reduction for ecotypes outside the habitat to which they are adapted and it should contribute to reproductive isolation [7]. Of particular interest are case studies in which the selective agents and the traits under selection have been identified [7], because then the mechanisms that link adaptation to the emergence of reproductive barriers can be studied and their importance assessed. For example, some populations of *Rhagoletis pomonella* fruit flies have recently switched from hawthorn to apple hosts and have adapted their phenology and fruit odour preference accordingly [8, 9]. In stickleback fish (*Gasterosteus aculeatus*), the repeated evolution of distinct trophic ecotypes within lakes is mirrored in the intricate, adaptive divergence of cranial morphology [10]. The intertidal snail *Littorina saxatilis* produces shells with different tickness and aperture in wave-exposed versus crab-rich sections of the shoreline, which provide better protection against dislodgement [11] and predation [12], respectively. These adaptations contribute to reproductive isolation, because each ecotype is less fit when transplanted into the respective other habitat [13, 14], because F1 hybrids are at a disadvantage [15, 16] and/or because the adaptations cause assortative mating [17–19]. A genomic dissection of such divergent traits can not only pinpoint the genomic regions under selection but also investigate genetic linkage between ecologically selected and mating traits [19] or, for example, explore the role of inversions in novel adaptations [20, 21]. Ecological divergence has long been assumed to generate barriers to gene flow in the hybrid zone of European fire-bellied toads [22], for which genomic resources have recently been developed [23]. Here we seek experimental evidence for adaptive divergence in tadpole traits as a first step in establishing a link between ecology and reproductive isolation.

In contrast to the very young taxa referenced above, the red-bellied toad (*Bombina bombina)* and the yellow-bellied toad (*B*. *variegata)* are more anciently derived [MRCA ~ 3.2 mya, 23]. Nevertheless, they interbreed wherever they meet and produce a wide range of recombinants in typically narrow (2–7 km wide) hybrid zones [24]. Classic cline theory [25] suggests that the mean fitness of intermediate hybrid populations is reduced by 42% compared to that of pure populations on either side [26]. There is experimental evidence for endogenous selection against hybrid embryos and tadpoles [27]. Profound ecological divergence is also strongly implicated. *B*. *bombina* reproduces in semi-permanent lowland ponds, such as flooded meadows and oxbows that fall dry in winter [e.g. 28]. *B*. *variegata* lays eggs in more ephemeral habitat (‘puddles’) and sometimes also in small steams [29], typically at higher elevations. Consequently, hybrid zones tend to be located at ecotones. There is a long list of taxon differences that presumably adapt both taxa to their current habitat [reviewed in 22]. For example, adult *B*. *variegata* have a relatively stronger skeleton, longer legs [30] and thicker skin [31], traits which presumably aid their frequent movements over land [32, 33], to track the ever-shifting mosaic of suitable breeding sites. Ponds are not only more predictable year-on-year but also allow for larger breeding aggregations. With their well-developed vocal sacs [34], male *B*. *bombina* produce much louder mating calls [35] and form choruses that can be heard from afar. A *B*. *variegata* male would likely be at a disadvantage if it joined such a chorus.

Here we focus on tadpoles, because they experience the strongest habitat contrast. Faced with the ever present risk of desiccation, *B*. *variegata* tadpoles hatch from relatively larger eggs, grow faster and metamorphose earlier [30, 36]. They also spend relatively more time swimming and feeding [37, 38]. The less active behaviour of *B*. *bombina* tadpoles should protect them against visually hunting predators in ponds [37]. These taxon differences reflect a general pattern across anurans. More permanent aquatic habitats have a greater abundance and diversity of predators [39–41]. Anuran species that reproduce in them have typically quiescent tadpoles [40, 42–44] that take longer to reach metamorphosis than those that breed in ephemeral sites [42, 43, 45]. More active tadpoles species are typically more susceptible to predation [42, 46–50]. This robust pattern has been interpreted as a trade-off [42, 45, 51, 52]: tadpoles can either be highly active and maximise food intake for fast growth and development in temporary habitats or avoid predators through quiescent behaviour in more permanent sites, but not both. Tadpole activity and growth rate covary as expected in evolutionary contrasts [53] but evidence for a causal relationship is mixed at the intra-specific level [54–59]. However, the trade-off between rapid growth and predator avoidance is almost universal [60] irrespective of whether activity is the mechanism that links the two. We therefore expect that *B*. *variegata* tadpoles are at greater risk of predation than *B*. *bombina*.

Nevertheless, *B*. *variegata* tadpoles do sporadically encounter predators such as newts, dragonfly and dytiscid larvae, water scorpions and salamander larvae [29]. Thus, tadpoles would maximise their chance of reaching metamorphosis by adjusting their phenotype to the local balance of risk. Indeed, a wide range of anurans display phenotypic plasticity in response to predators. Almost invariably, tadpoles reduce their activity level when they perceive chemical cues that indicate predator presence [45, 61, 62]. This response can occur within minutes [63]. Longer term exposure to such cues can cause an increase in refuge use [64, 65], alter larval morphology [62], prompt the development of colour spots on the tail [66, 67], and slow growth and development [68, 69]. These effects are seen across venues from the laboratory to the field [70, 71].

The commonest and most closely analysed morphological response to predators is the development of a relatively deeper tail fin [43, 44, 55, 62], usually in combination with a shorter tail and a shallower body. A deeper tail may facilitate rapid escape (faster burst swimming speed) or lure attacks away from the more vulnerable body. While there is evidence that natural variation in tail shape affects tadpole swimming performance [50, 72], studies to date have given stronger support to the lure hypothesis [73, 74]. Irrespective of the particular mechanism, there is evidence that predator-induced phenotypes are eaten less than predator-naïve ones [75–78] and that survival is correlated with measured traits that show a plastic response [78–80]. A recent study of *Rana temporaria* [81] makes a particularly strong case for adaptive phenotypic plasticity in both behaviour and morphology, including an assessment of the variability in predation risk within and between ponds and over time that could bring about such a flexible strategy.

Both *B*. *bombina* and *B*. *variegata* respond to the presence of predators with reduced activity [37, 38] and by developing a relatively deeper tail fin [82, see also 62]. Exposure to chemical predation cues slows their growth and development under laboratory conditions [82]. While there is at present no evidence that the induced phenotypes are better protected from predators, they should be used for a fair comparison of relative predation risk, because tadoles would develop with predators in nature. A previous study using predator-naïve tadpoles, while showing the expected result, could therefore not fully settle the issue [37]. In a second laboratory experiment with predator-induced tadpoles, a strong population effect overwhelmed any taxon differences [82]. In both cases, significant effects were observed only in the earlier of two sets of predation trials, which suggests that relative susceptibility may change over time.

Here, we carried out laboratory experiments that improve on the design of the previous two studies by using predator-induced phenotypes throughout, by increasing the level of replication within each taxon and by estimating relative predation risk over a four-week period, from hatching to the time when the first *B*. *variegata* tadpoles start to metamorphose. Dragonfly larvae were used as predators, because they are ubiquitous in ponds and also found in puddles. We also assessed the contributions of activity, body size and tail shape to the observed differences in predation risk. Our experiments confirmed our expectation that pure *B*. *variegata* suffer a higher predation rate than pure *B*. *bombina* due to a higher activity level. In the discussion, we argue that elevated activity contributes to the gene flow barrier across the hybrid zone which keeps the two gene pools from merging into one.

## Materials and methods

### Animal collections, pairing scheme and toad care

Adult toads were collected from Moravia (Czech Republic) where the Carpathian lineage of *B*. *variegata* meets the Southern lineage of *B*. *bombina* [83]. The experiment was carried out in 2017. Starting in early May, aquatic habitats were visited regularly (14 known *B*. *bombina* sites and 19 known *B*. *variegata* sites). Due to an unusually cold spring, toads did not appear in appreciable numbers until early June and were then promptly collected to ensure that females had not yet laid eggs. Collection sites (Table 1) were located at least 6 km from known areas of hybridisation and deemed 'pure' based on the taxon-specific spot pattern on the toads' bellies [22]. For each taxon, minimum and maximum distances between sites were 1.5 and 43.6 km (*B*. *bombina*) and 0.7 and 14.9 km (*B*. *variegata*). *B*. *bombina* sites were ponds with a minimum width of 5 m and a maximum depth greater than 1 m. They all contained predators (incl. *Aeshna cyanea*, *Natrix natrix*, fish) and, with one exception (site E), they had abundant aquatic vegetation. *B*. *variegata* sites were either water-filled wheel ruts (max. depth < 30 cm) or in one case (site 2) the shallow end of an abandoned swimming pool. They were devoid of aquatic vegetation and three of nine sites contained no predators at all (see S1 Table for details).

**Table 1:** Collection sites and pairing scheme.

Taxon	Site	Coordinates (N)	Coordinates (E)	Family indices
*B*. *bombina*	E	49°15´10''	15°41´35"	13
	H	49°13´30.6"	16°02´53"	18, 19, 20
	K	48°51´41.078"	15°43´39"	15, 16
	M	49°13´59"	16°03´50.5"	11, 12, 17
*B*. *variegata*	1A	49°06´27.6"	17°13´52.3"	1, 2, 7(*m*)
	1B	49°06´25.5"	17°13´51.9"	6, 7(*f*)
	2	49°08´26"	17°21´51"	3
	5	49°05´59.82"	17°13´11"	10(*f*)
	6	49°10´04.6"	17°22´25.8"	8
	16	49°05´11"	17°12´43"	4(*m*)
	17	49°05´23.5"	17°13´26.7"	4(*f*)
	19	49°05´52.3"	17°13´25.2"	9(*m*), 10(*m*)
	20	49°06´00.1"	17°13´33.8"	9(*f*)

In four *B*. *variegata* families, the parents came from different sites: *m* and *f* in parentheses indicate the origin of the male and the female, respectively.

Random numbers were drawn to pair males and females within sites whenever possible. However due to unequal sex ratios per site, four *B*. *variegata* pairs involved toads from separate, but nearby sites (Table 1, maximum distance between sites per pair: 0.96 km). Each pair was housed in a 52 x 35 cm plastic box with 7L of well water, a polystyrene island and three pieces of plastic aquarium plants as support for egg batches. As a precaution against the potential spread of *Batrachochytrium* infection, all waste water from the entire experiment was heated to 100° C and treated with UV (Eheim UV reflex UV800) before disposal to kill any fungal spores.

On 20 June, a belly swab was taken from each toad to test for *Batrachochytrium dendrobatidis (Bden)* and *B*. *salamandrivorens (Bsal)* infection. The former pathogen is widely distributed in the Czech Republic, but so far no population declines have been observed [84]. The qPCR followed the protocol of Blooi et al. [85] using a Roche Light Cycler480. No evidence of either infection was found. We note that samples in the same qPCR run but from an unrelated study scored positive for *Bden* infection. The adults were subsequently returned to their exact collection sites.

### Predators

We used *Aeshna cyanea* larvae as predators. This dragonfly is widely distributed across Europe and was present in most of the habitats we surveyed (see above). Two hundred larvae were collected from one site near Jihlava (Vysočina County) and were kept in groups of 15 in 50L plastic boxes. They were fed every other day with Tubifex worms before the predation trials started and with tadpoles thereafter.

### Oviposition

To induce reproduction, 250 I.U. of human chorionic gonadotropin, dissolved in 0.2 ml of amphibian Ringer solution, were injected into the lymph sac of an outer thigh of both males and females on 6 June. Most pairs produced egg clutches overnight. Over the following two days, 30–40 eggs per family were transferred into the rearing set-up (see below). The remaining offspring were raised in the oviposition boxes, with weekly water changes. They were used as food for dragonflies in order to condition the water in the rearing set-up. The adults were moved to new boxes of the same size. Tadpoles hatched on 12–13 June. In the following, we refer to 13 June as day 0 of the experiment.

### Tadpole rearing

**Fig 1:**
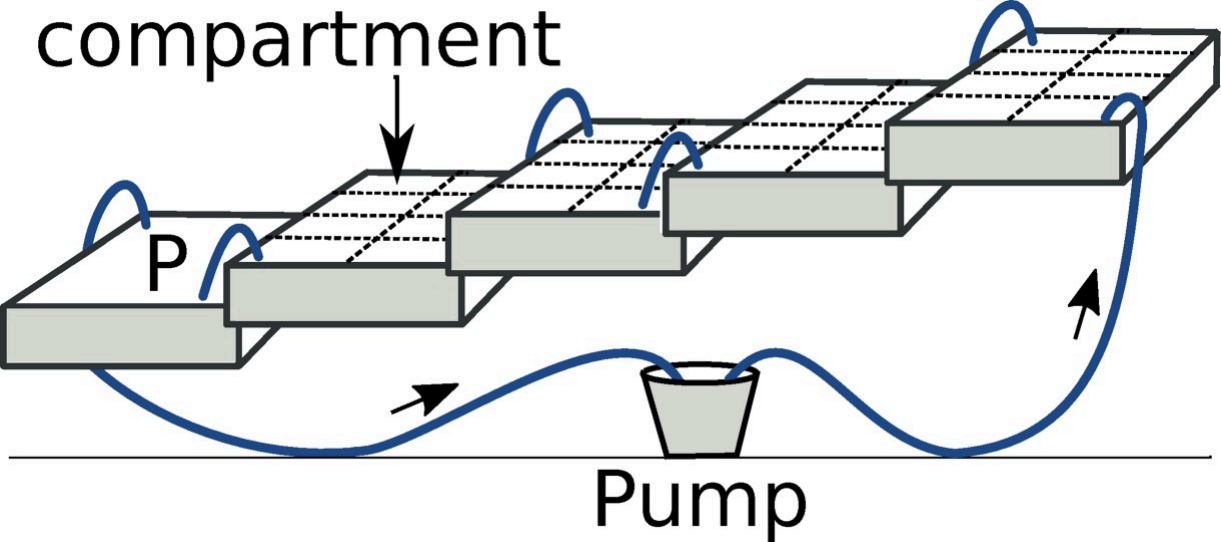
Schematic of one gravity flow circuit. At the start of the experiment, each of the 32 compartments contained 10 hatchlings from a single family. A dragonfly was regularly fed non-experimental tadpoles in the bottom tub (P) so that chemical predation cues were present throughout tadpole development. Blue lines indicate silicone tubing and arrows show the direction of flow.

We constructed two gravity flow circuits (Fig 1). For each of them, five plastic tubs (52 x 37 x 16 cm) were interconnected with silicone tubing such that water could be pumped from a 20 L bucket at floor level into the top tub and then descend stepwise via the other four tubs before returning to the bucket. Inflows and outflows in each tub were positioned diagonally opposite to maximise mixing. Each tub held 27L of water at a depth of 14 cm. In order to maintain uniform water quality, the pumps (Eheim StreamON+ 2000) were operated 24 hours per day at a low flow rate and per circuit 7 L of water were replaced daily. Throughout the experiment, water temperature was 22–24°C and the air temperature was 24–26°C. All water changes for adults and tadpoles were carried out with well water heated to 22°C and sterilised with UV (Eheim UV reflex UV800).

The upper four tubs in each circuit were subdivided with fibreglass insect mesh into eight rearing compartments (size: 18 x 11.5 x 16.5 cm, 64 compartments in total). A plastic aquarium plant was added to each compartment for structural complexity. At the start of the experiment, each compartment held 10 hatchlings from a single family. Families (nine per taxon) were distributed over either three or four compartments in total and either one or two compartments in each circuit. Exactly half of the compartments in each circuit were assigned to each taxon. Placement of families within circuits was decided by random draws. Tadpoles were fed boiled nettle (*Urtica dioica*) leaves *ad libitum* and uneaten food was removed every day.

In order to simulate predator presence, we let chemical cues of tadpole predation circulate from day 0 onwards. For this, we used the fifth tub in each circuit to feed 1–2 non-experimental tadpoles (depending on size) to one dragonfly larva every other day. Tadpoles of both taxa were used for this in equal proportions.

### Predation trials

Predation trials started on day 2 when the tadpoles were active. They were carried out in two plastic boxes subdivided with fibreglass insect mesh into four sections (26 x 26 x 18 cm), each with two plastic aquarium plants (akin to *Myriophyllum*), placed into diagonally opposite corners. They contained a 1:1 mixture of well water and water from the rearing circuits. For each trial, four tadpoles (2 *B*. *bombina*, 2 *B*. *variegata*) were placed into one section and allowed to acclimatize for 30 min. A dragonfly that had not been fed on the previous day was then added and the trial was stopped when the first tadpole had been eaten (maximum duration: 45 min). The identity of the missing tadpole was determined from digital photographs of the animals taken before the trial (see below). The survivors were returned to their rearing compartments. The trials were recorded with a Microsoft LifeCam VX-2000 video camera suspended over each plastic box.

Predation trials were carried out on 18 days as follows: two consecutive experiment days were followed by one ‘off-day’ (1 exception); this pattern was repeated nine times until day 29 post hatching, when the first *B*. *variegata* tadpoles entered metamorphosis. There were 20 trials per day between 10:00 and 15:00 hours. A custom Perl script was used to haphazardly draw the composition of each trial per day such that (a) four different families were represented, (b) one tadpole per taxon came from circuit 1 and the other from circuit 2 and (c) each rearing compartment contributed at least one tadpole on a given day. However towards the end of the experiment, a few rearing compartments were empty and the contribution of others had to be increased accordingly. No individual tadpole or dragonfly was used more than once per experiment day.

### Morphometrics

Digital images were taken by placing a tadpole into a glass cuvette and photographing it from the side with a Canon EOS 400D camera (lens: Canon EF 28-90mm). tpsDIG2 (F. James Rohlf) software was used to take the following measurements from these images: total length, tail length, body height and maximum tail height.

### Behaviour

From the video footage of the predation trials, we recorded the behaviour of a focal tadpole during one 30 s interval per trial for half of the trials on a given day. A custom Perl script provided the pseudo-random start point of the observation interval and allowed behaviour (swimming, feeding, resting) to be recorded with separate key strokes. From one trial to the next, we alternated between a *B*. *bombina* and *B*. *variegata* tadpole. Taxon identification was based on size in the early trials. Morphological differences were used as soon as they became apparent. Footage from the first two days was not used, because the taxa could not be reliably distinguished. We discarded all observation intervals that overlapped with a predator attack. We could not determine whether the focal tadpole survived the trial, because that would have increased the data collection time roughly 10 to 20-fold. Moreover, it was difficult to track an individual over several minutes, especially when it temporarily disappeared together with others under a plastic plant. We also recorded for 68 predator attacks (four on each of 17 experiment days) whether they were initiated by and directed towards an actively swimming tadpole.

### Permits

Collection permits were granted by the relevant regional offices (South Moravia and Zlín) of the Czech Ministry for the Environment. The experimental protocol was approved by the ethics committee of the Institute of Vertebrate Biology, CAS, permit no. 15/2016.

### Statistical analyses

#### Estimates of predation risk

We estimated predation risk per rearing compartment (= the unit of observation, containing offspring of one family) as the number of tadpoles eaten divided by the number of valid trials in which that compartment was represented. This figure subsumes the ontogeny of tadpoles in terms of changes in size, body shape, behaviour and, possibly, experience in predators avoidance. Recall that surviving tadpoles were returned to their rearing compartment and were likely picked again in later predation trials. Just as in nature and in the classic mesocosm studies [46, 55, 86], in which tadpole cohorts interacted with predators for a substantial part of larval development, older tadpoles were likely to have survived previous predator encounters. Specific to our design is that we staged these encounters and collected additional data about each one of them.

Especially with a small number of trials, our estimate of predation risk may be biased if tadpoles from a certain compartment happened to be assigned to trials with, say, particularly well defended congeners. There is a range of statistical methods to estimate an individual's skill from a limited number of contests with opponents of varying strengths (as e.g. in chess), starting with the model of Bradley and Terry [87]. However, none of these match our trial design of a single winner (or rather: looser) and a 'tie' for the other three.

We therefore checked for biases due to a limited number of trials as follows. Each trial *k* involves tadpoles from four (of 64) compartments. The risk context *rc*_ik_ of the tadpole from compartment *i* (1 ≤ *i* ≤ 4) is the mean of the predation risks *r*_j_ of the other three compartments that are represented:

$${rc}_{ik}=\sum_{j=1,j\neq i}^{4}\frac{r_{j}}{3}$$

For a given compartment *m* (1 ≤ *m* ≤ 64), the average over its associated *rc*_ik_ estimates in valid trials is the mean risk context, *rc*_m_. If our estimates of predation risk, *r*_m_, are unbiased, then the estimates of risk context from the observed dataset should be very similar to those from a much larger set of trials. To test this, we haphazardly drew compartments for another 2550 trials with the same script as before and used these ‘mock trials’ to re-compute risk contexts using the *r*_m_ estimates from the actual experiments.

We used linear mixed models to investigate the effect of taxon on predation risk and included ‘circuit’ and ‘family’ terms to properly represent the non-independence of compartments. Circuit was treated as a fixed effect, because it had only two levels. We did not include a population term in the model, because populations were unevenly represented and some families had parents from different sites. Any differences between populations should appear as family effects. For these, we fitted not only random intercepts but also allowed the effect of ‘circuit’ to vary among families (random slopes). Model were fitted with the R module *lme4* [88, 89]. We used the ‘REML = FALSE’ option in order to compare models via a likelihood ratio test.

#### Morphological correlates of survival

Logistic models were used to investigate the effects of taxon, tadpole size and tail shape on survival per trial. The input was generated by sorting all valid trials in chronological order and picking from them alternately (a) a surviving tadpole at random or (b) the non-surviving tadpole. For the entire dataset, the four morphological measurements were log-transformed to improve normality and entered into a principal component analysis (PCA). We treat the first principal component as a measure of tadpole size. From a regression of log(tail height) on PC1 we extracted the residuals as a measure of relative tail height (≈ tail shape). To ensure shared allometry between the taxa, we inspected pairwise plots of the four measured variables [90]. For the focal tadpole per trial, PC1 and residual log(tail height) were expressed as a deviation from the trial mean. All analyses were carried out in R v.3.4.4 [91].

## Results

### Taxon differences in predation risk

**Fig 2:**
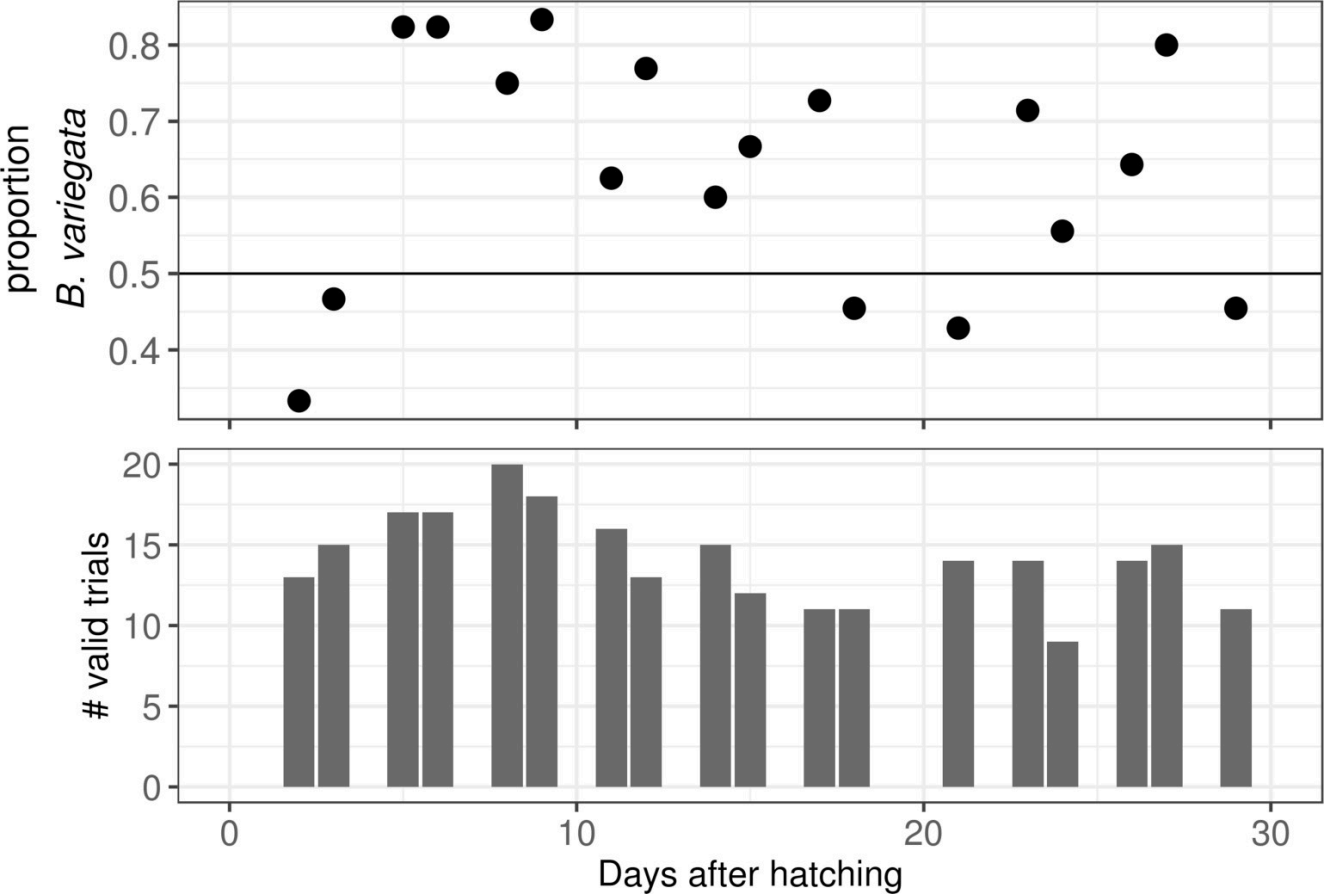
Relative predation risk and number of valid trials per day. A. The proportion of tadpoles consumed that were *B*. *variegata*. Tadpole age (days after hatching) is plotted on the x-axis. Each data point gives the proportion for one experiment day. B. The number of valid trials (out of 20) per experiment day.

We assessed relative predation risk over a four-week interval. Our experiments started two days after hatching when tadpoles were uniformly at Gosner [92] stage 23. On the last experiment day, *B*. *bombina* tadpoles were mostly at stages 35–36 whereas the commenest *B*. *variegata* stages were 39–40 with some individuals entering metamorphosis (stages 42+). In total, 360 trials were conducted. Of these, no tadpole was eaten in 97 trials, more than one was eaten in five trials and in three trials the two taxa were not evenly represented. Discarding these, 255 valid predation trials remain. The proportion of valid trials in which a *B*. *variegata* tadpole was eaten was 0.65. The taxon difference was particularly strong for 5–12 day old tadpoles, when around 77% of the tadpoles eaten were *B*. *variegata* (Fig 2A). There was also a slightly higher risk for tadpoles from circuit 1 as opposed to circuit 2 (proportion = 0.56). This effect was largely restricted to *B*. *bombina*. The number of trials in which no tadpole was eaten was smaller in the first half of the experiment (tadpole age: 2–14 days, *n* = 31) than in the second half (tadpole age: 15–29 days, *n* = 66, Fig 2B). The mean durations of valid trials were 9.9 min (fist half) and 13.8 min (second half).

**Fig 3:**
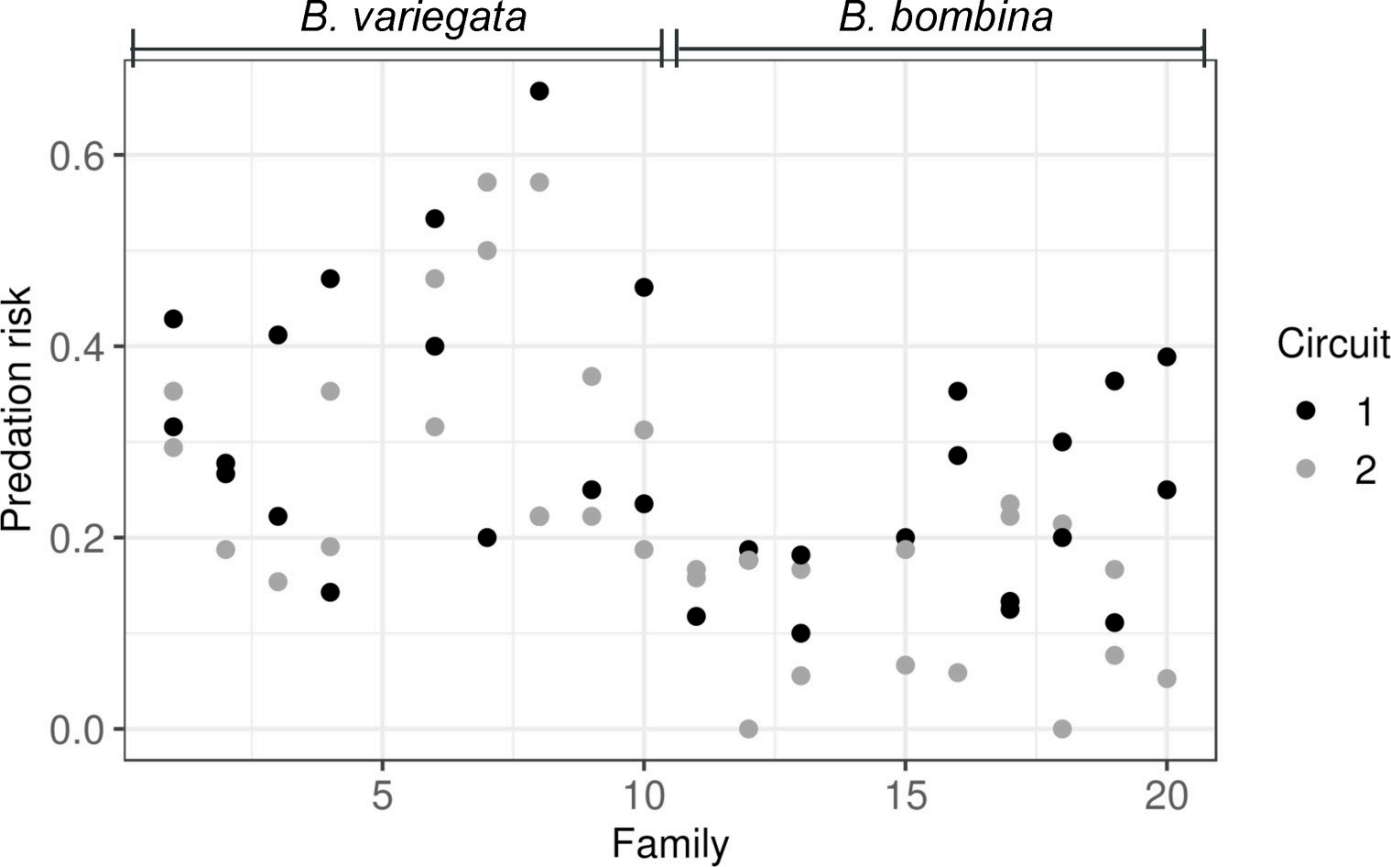
Predation risk per rearing compartment arrayed by family. *B*. *variegata*: families 1–4 and 6–10, *B*. *bombina*: families 11–13 and 15–20.

Predation risk was computed per rearing compartment as the number of tadpoles eaten during valid predation trials over the number valid trails in which this compartment was represented. The observed risk contexts (as defined in the Materials and Methods) closely matched those re-computed for a much larger number of hypothetical trials ($\bar{n}\mathrm{per}$ compartment = 159.9): for 56 of 64 compartments the observation deviated by less than 10% from the expectation (max. deviation 14%) and in all cases it was well within one standard deviation of the prediction (S1 Fig). We therefore use predation risk per rearing compartment as the response variable in the following analyses (Fig 3).

The full linear mixed model (Table 2, model 1) included taxon, circuit and family (random effect, nested within taxon). We allowed ‘circuit’ to affect each family differently (random slopes). As expected, there was an increased risk for *B*. *variegata* (*β* = 0.2069 ± 0.0357) and a slightly decreased in risk in circuit 2 (*β* = -0.0814 ± 0.0376). Family effects (intercepts and slopes) accounted for 13.9% of the variance. The reduced model 2 without the ‘taxon’ term gave a much worse fit to the data, resulting in a highly significant likelihood ratio test (χ^2^ = 19.12, 1 df, *p*-value = 1.228 x 10^−05^). There is thus clear evidence that *B*. *variegata* tadpoles were at relatively greater risk of predation by *Aeshna cyanea* larvae. Nevertheless, a large amount of unexplained variation remains (Fig 3).

**Table 2:** Linear mixed models of predation risk per rearing compartment.

No.	Model	logLikelihood
1	asin_risk^a^ ~ taxon + circuit + (1 + circuit | family)^b^	33.163
2	asin_risk ~ circuit + (1 + circuit | family)	23.603

^a^arcsine(sqrt(risk)).

^b’^family’ is a random effect nested implicitly within ‘taxon’. ‘circuit’ is allowed to affect each family differently (random slopes).

### Tadpole size and shape

**Fig 4:**
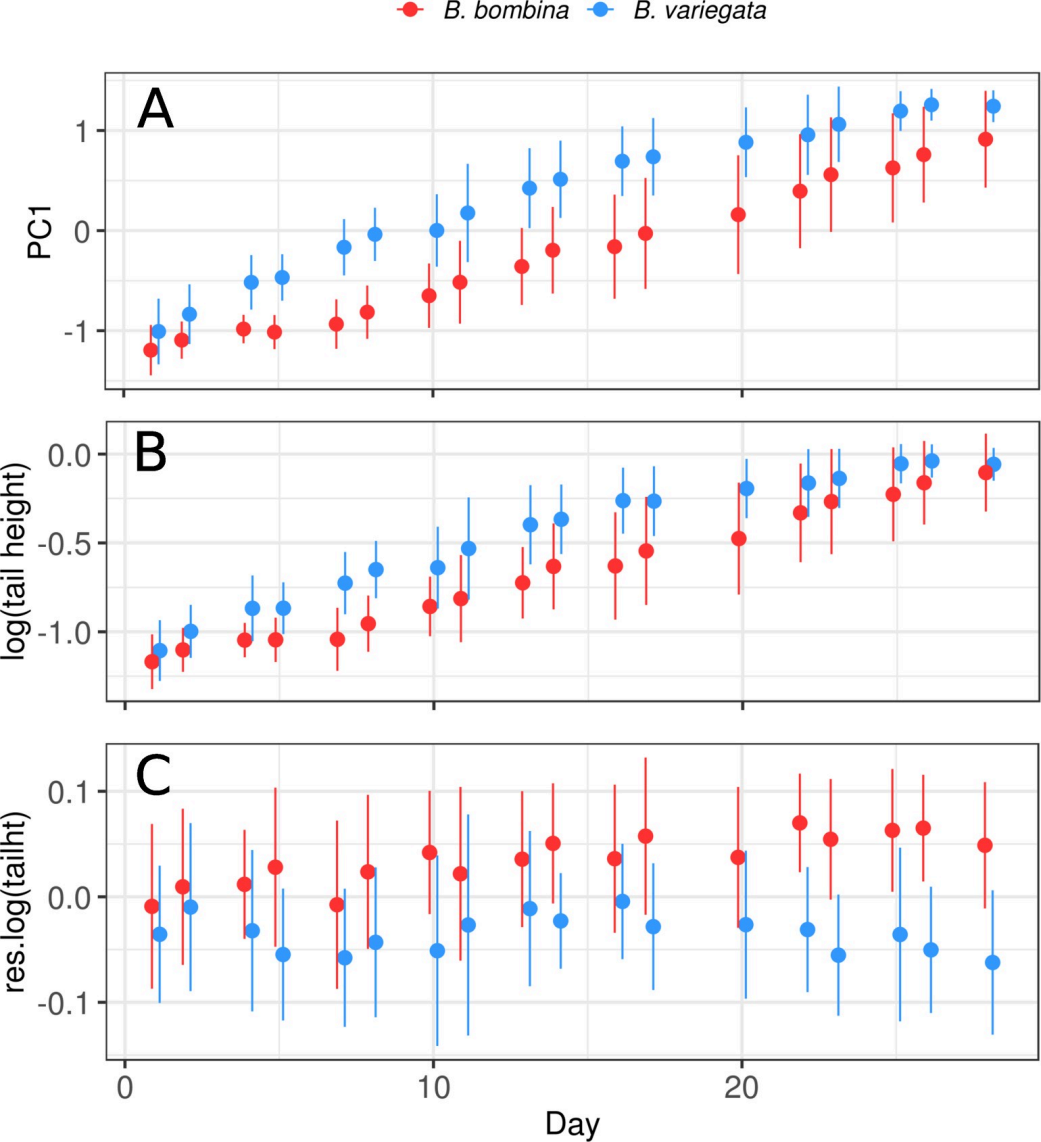
Morphological development by taxon. Means and standard deviations by taxon for each of three descriptors of morphology. A. PC1 = 1. principal component ≈ body size, B. log(tail height), C. residual(log(tail height)) = residuals of a regression of log(tail height) on PC1 ≈ size-corrected tail height.

Throughout the experiment, *B*. *bombina* was on average smaller with a shallower tail (Fig 4A and 4B). But after correction for body size, *B*. *bombina's* tail fin was relatively higher than that of *B*. *variegata* (Fig 4C). Note that this is a difference between two types of predator-induced phenotypes. It does not tell us about the strength of the plastic response in either taxon, because predator-naïve tadpoles were not part of this study. Our shearing approach to correct for body size is validated by the shared allometry in both taxa (S2 Fig). If size and/or tail shape are functionally important, we would expect to see within-taxon correlations with survival as well. The logistic regression analysis (Table 3, see Materials and Methods for details) confirmed the significantly lower survival of *B*. *variegata* and also showed a strong interaction of tadpole size (PC1) and taxon. There were no significant effects associated with relative tail height.

**Table 3:** Coefficients of the logistic model of survival.

	Estimate	Std. Error	z value	Pr(>|z|)
(Intercept)	0.9110	0.3036	3.001	0.00269**
taxon (*B*.*v*.)	-1.0405	0.4161	-2.501	0.01240*
PC1	1.9817	0.6327	3.132	0.00173**
resid(LTH)^a^	5.1895	3.6139	1.436	0.15100
taxon*PC1	-2.4135	0.8963	-2.693	0.00709**
taxon*resid(LTH)	-2.2078	4.7176	-0.468	0.63978

Survival is coded as 1 (yes) and 0 (no). Morphological measurements were expressed as deviations from the trial mean. See legend of Fig 4 for an explanation of the independent variables.

^a^residual(log(tail height)). Significance levels: α < 0.05 (*), α < 0.01 (**).

**Fig 5:**
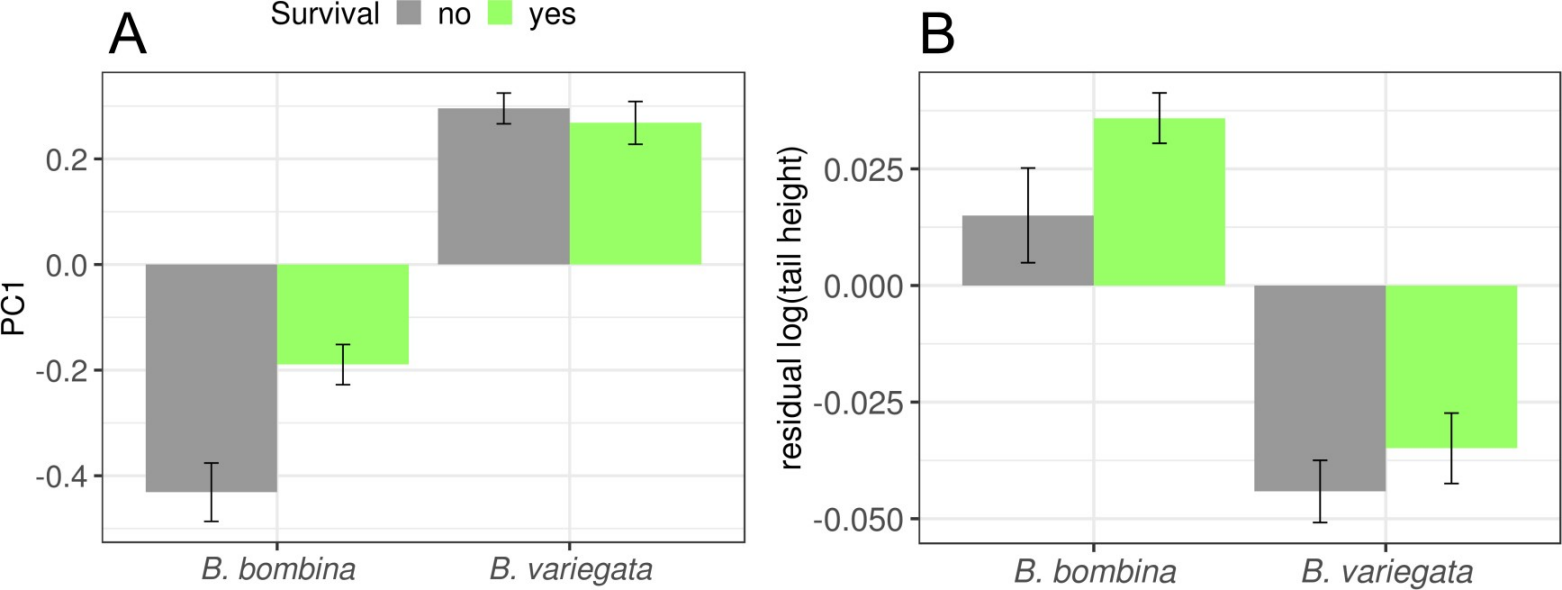
Means of body size and tail shape by taxon and survival. Plotted are the fitted means from the logistic regression analysis for PC1 (A) and residual log(tail height) (B). Error bars indicate one standard error of the mean.

Fig 5A illustrates that larger *B*. *bombina* tadpoles survived better than smaller ones (t = 3.5993, df = 85.589, p-value = 0.0005). But this effect is not consistent across the whole dataset. *B*. *variegata* tadpoles are overall larger and more at risk of predation, whereas smaller size is associated with greater risk in *B*. *bombina*. There is therefore no evidence that a general preference of *Aeshna* larvae for larger prey causes higher mortality in *B*. *variegata* tadpoles. The analogous plot for relative tail height hints at an advantage for higher tail fins (Fig 5B), but the present dataset provides no statistical support for this hypothesis.

### Tadpole activity

During the 30 s observation intervals, both taxa spent most of their time either resting or, seemingly, feeding. No food was provided in these trials. So, the tadpoles' persistent scraping of surfaces with their mouthparts, especially during the first half of the experiment, probably provided little, if any nutrition. Swimming typically occurred in short bursts of a few seconds separated by inactive intervals. Ninety-two percent of recorded predator attacks were directed at actively swimming tadpoles.

**Fig 6:**
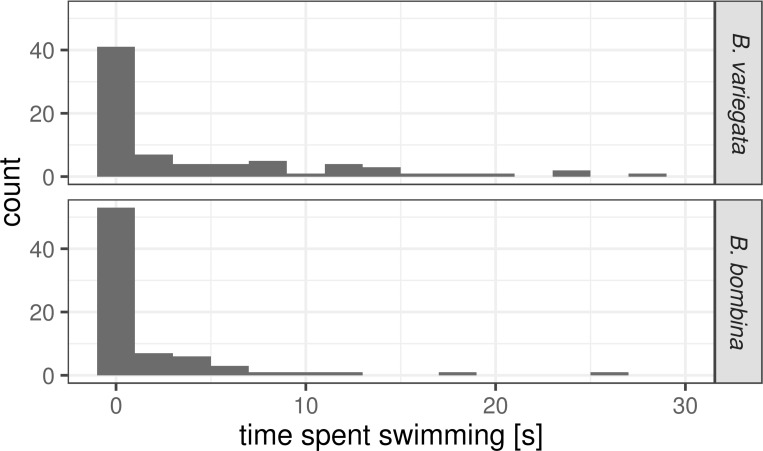
Distribution of the time spent swimming by taxon. Each observation interval lasted for 30 s. Number of intervals: 75 and 74 for *B*. *variegata* and *B*. *bombina*, respectively.

The distributions of the time spent swimming were strongly skewed to the right in both taxa (Fig 6). As expected, *B*. *variegata* spent more time swimming than *B*. *bombina* (means: 4.27 and 1.78 s, respectively; unpaired Wilcoxon rank sum test, W = 2234.5, p-value = 0.0096). Focal tadpoles were chosen based on their clear visibility during the 30 s observation interval. Their survival status is unknown (see Materials and Methods). We can therefore not investigate within-taxon correlations between activity and survival.

## Discussion

In our experimental setting, tadpoles of the puddle breeder *B*. *variegata* are at greater risk of predation from dragonfly larvae than those of *B*. *bombina* that develop in semi-permanent ponds. This result confirms our expectation based on similar species comparisons [42, 43]. It also agrees with the findings of Kruuk and Gilchrist [37] who conducted predation trials with larger groups of predator-naīve *B*. *bombina* and *B*. *variegata* tadpoles that could only adjust their behaviour. Here, we allowed tadpoles to develop a more complete predator-induced phenotype. Nonetheless, the survival difference between the taxa persisted and it was particularly strong during days 5–12 (Fig 2A). In previous mesocosm experiments with pure and hybrid *Bombina* and free-ranging dragonflies, the overall predation rate declined sharply from day 15 onwards even though tadpoles continued to be attacked [93, see similar results for *Rana*, 94]. Thus, the days with the highest total predation risk overall may coincide with a particularly strong disadvantage for *B*. *variegata*.

The increase in no-predation trials in the second half of the experiment is consistent with the mesocosm observations. The overall proportion of such trials (0.27) was high in relation to comparable studies. In our previous *Bombina* study [82] using the same design (4 tadpoles, 1 dragonfly), all trials resulted in predation. But the trial compartments were only half the size and so presumably led to more predator-prey encounters. McCollum and Van Buskirk [75] exposed *Hyla versicolor* tadpoles (two per trial, 1.5 times larger boxes) to predators for up to 500 min. Both tadpoles survived in 7% of cases. Our trials may have led to a roughly similar outcome, had they been extended from 45 to 500 min. The fact that all tadpoles managed to avoid predation in a quarter of cases suggests that we did not create a completely ‘hopeless’ and thus unrealistic situation but instead one that even the more susceptible taxon could negotiate successfully. Many predator attacks were unsuccessful, because tadpoles could escape via sprints or agile movements through the vegetation.

Many tadpoles were used in more than one trial. Later trials may therefore have involved individuals that benefited from experience gained and/or were inherently better at avoiding predators. We believe that this resembles the situation in nature more closely than the alternative of shielding tadpoles from predator encounters until the day of their one experimental test. As pointed out by Melvin and Houlahan [95], typical tadpole mortality in nature (approx. 95% according to their review) may be non-random with regard to traits under study. The generally much more benign conditions in the laboratory may thus produce experimental cohorts that are not representative of the population of origin. Moreover, we specifically set out to integrate predation risk over a substantial part of larval ontogeny with all its dramatic changes. We consider any gain in experience to be part of this process. Nonetheless, this close-up view of predator-prey interactions is a piece in a mosaic that should be complemented with experiments in semi-natural settings and using additional predator species.

If *B*. *bombina* and *B*. *variegata* were reproductively isolated, these results would address the question why *B*. *variegata* reproduces only rarely in semi-permanent, predator-rich ponds [96]. Whether or not *B*. *bombina* tadpoles are present in these habitats may not matter, because there is little evidence for competition among tadpoles in ponds [41, 43, 46]. Predators tend to keep tadpole numbers sufficiently low so that competition is negligible. *B*. *bombina* then simply provides a benchmark for a successful 'pond strategy'. *B*. *variegata*'s greater susceptibility to predation shows that its tadpoles fall short of this benchmark even when they display the predator induced phenotype. Other traits such as relatively fewer eggs per clutch [36] may also limit *B*. *variegata's* reproductive success in ponds.

Hybridisation shifts the focus away from a comparison between distinct types and towards selection on traits in recombinants. These genotypes predominate in *Bombina* hybrid zones, while F1 individuals are very rare, if present at all [24, 26]. A hybrid genome consists of blocks of varying sizes that are inherited from one or the other species. Within the hybrid zone and with each round of recombination, these blocks get smaller, while the influx of pure genomes from the periphery introduces new large blocks [97]. The chance of, say, a *B*. *variegata-*derived block to cross into the *B*. *bombina* gene pool depends on how it affects the fitness of its carriers along the way. A genetic variant that reduces fitness in ponds is likely to be eliminated from this habitat together with the entire *B*. *variegata*-block in which it resides. It is in this sense that such variants act as barriers to gene flow [98].

This reasoning extends to adaptively diverged traits, no matter whether the taxon difference is based on one locus or on several unlinked, additive loci: any maladapted *B*. *variegata* variant would increase the probability that the block in which it resides will be eliminated from ponds [99]. Smooth clines of six quantitative traits mapped across the hybrid zone near Pešćenica, Croatia, indeed suggested an largely additive genetic basis [30]. For highly integrated compound traits, the gene flow barrier may be further strengthened if intermediate hybrids have reduced fitness due to mismatched phenotypes. This has been demonstrated in F2 crosses of sticklebacks (limnetic x benthic ecotypes), where numerous quantitative trait loci underlying the trophic adaptations are known: F2 hybrids with certain intermediate genotypes expressed mismatched phenotypes and grew more slowly [100]. Overall, the contribution of ecological divergence to the maintenance of distinct taxa should increase as the list of traits under selection gets longer [101, 102].

Prey activity alerts visually hunting predators and correlates strongly with tadpole predation rates in inter-specific comparisons (see Introduction) and also within species [75, 103]. Our findings confirm this pattern. Because the taxon difference in activity is seen in animals raised under uniform laboratory conditions, it should have a genetic basis. We do not know how many loci are involved but we expect that each *B*. *variegata*-derived genetic variant that crosses the hybrid zone and increases activity in a *B*. *bombina* genetic background is selected against. It thus reduces gene flow at genetically linked loci.

For the sake of argument we consider two alternative scenarios. There may be a hybrid genotype with an activity level that increases fitness over that of either pure taxon. This type of hybrid might then contribute to an increase rather than a reduction in gene flow. However, we do not find it likely that activity has an intermediate optimum that has not yet been reached in either taxon. It could also be that a high-activity variant is closely linked to a universally favourable mutation that arose in *B*. *variegata*. The mean fitness of blocks including both variants might then be higher than that of their *B*. *bombina* counterparts, generating a conduit of gene flow in this genomic region. But the effect of the activity variant would still be to slow the introgression of such a block into the *B*. *bombina* gene pool and the ‘pull’ of the favourable variant would cease as soon as recombination separates the two [99]. On balance, these considerations do not alter our prediction that higher activity most likely contributes to the reduction in gene flow across the hybrid zone.

There was no evidence that relatively higher tail fins caused better survival in either taxon. The large variances that rendered differences between group means (taxon, survival) non-significant (Figs 4C and 5B) may reflect heritable variation among families in the amplitude and type of morphological plasticity [104]. This trait warrants further study using more detailed morphometrics [72]. Finally, our analyses disproved the hypothesis that an overall preference of *Aeshna cyanea* larvae towards larger prey contributed to *B*. *variegata's* greater mortality.

Is higher predation risk in *B*. *variegata* a direct consequence of adaptation to ephemeral habitat? Population studies have shown that successful metamorphosis can be restricted in a given year to less than 50% of sites in which eggs have been laid (depending on annual rainfall) and that desiccation is the main cause of reproductive failure [105–107]. This is reflected in the growth rate of *B*. *variegata* tadpoles which was the fastest in a set of 15 European and North American anurans [68]. In contrast to the situation in ponds, intra-specific competition should be commonplace for *B*. *variegata* as tadpoles grow and puddles shrink [108]. The typically high activity of tadpoles growing in this type of habitat supports this [42, 109]. In the laboratory, large *B*. *variegata* tadpoles displace smaller conspecifics from food patches with their powerful tail movements [110]. Not only is the abundance and diversity of predators that *B*. *variegata* encounters lower than in ponds [37, see also 39–42], sometimes these early-successional sites are predator-free. This suggests a hierarchy of risk: rapid development takes priority whether or not there are predators. They add to the already considerable unpredictability of reproductive success. Bet-hedging may be a way to sidestep an unresolvable trade-off [111, 112]. Adult *B*. *variegata* can live up to at least 15 years in nature [29, but see: 113] and females are known to lay eggs repeatedly over their lifespan [29, 112].

There may be aspects of *B*. *variegata*’s anti-predator strategy that remain to be discovered. *B*. *variegata* frequently develops dark tail spots that may divert attacks away from the body, whereas *B*. *bombina*'s tail fin is always uncoloured [114]. Moreover, the animals may not have been able to express some behavioural strategies in our experimental setting. Nonetheless, our results show that high activity in *Bombina* tadpoles is associated with higher predation risk that causes relatively higher mortality in the more active taxon, *B*. *variegata*, even when the tadpoles express a predator-induced phenotype. Population genetic theory predicts that *B*. *variegata* alleles underpinning high activity will be selected against in typical *B*. *bombina* breeding habitat and should therefore act as barriers to gene flow across the hybrid zone.

In the context of ecological speciation (cf. Introduction), the remaining question is whether these taxon differences reflect selection towards different phenotypic optima. Given that quiescent behaviour benefits *B*. *bombina*, what are the benefits of high activity for *B*. *variegata*? If indeed rapid growth and development in a competitive environment require active tadpoles, then the classic trade-off applies: activity mediates two mutually exclusive strategies [42, 45, 46, 51, 52]. In *Bombina*, this could be tested not just by comparing the pure taxa, but also by investigating phenotypic and genetic covariances in natural hybrids and by mapping the component traits in the *Bombina* genome [23].

## Supporting information

S1 Fig Risk context per rearing compartment. (PDF)

S2 Fig Pairwise plots of morphological traits. (PDF)

S1 Table Ecological features of the collection sites. (PDF)
