# Supplementary material for: Tadpoles of hybridising fire-bellied toads (B. bombina and B. variegata) differ in their susceptibility to predation
Source: PLoS One. 2020 Dec 7;15(12):e0231804. doi: 10.1371/journal.pone.0231804 (PMC7721483; doi:10.1371/journal.pone.0231804)
Supplement: S1 Fig — (PDF) [file pone.0231804.s001.pdf]

S1 Fig. Risk context per rearing compartment.

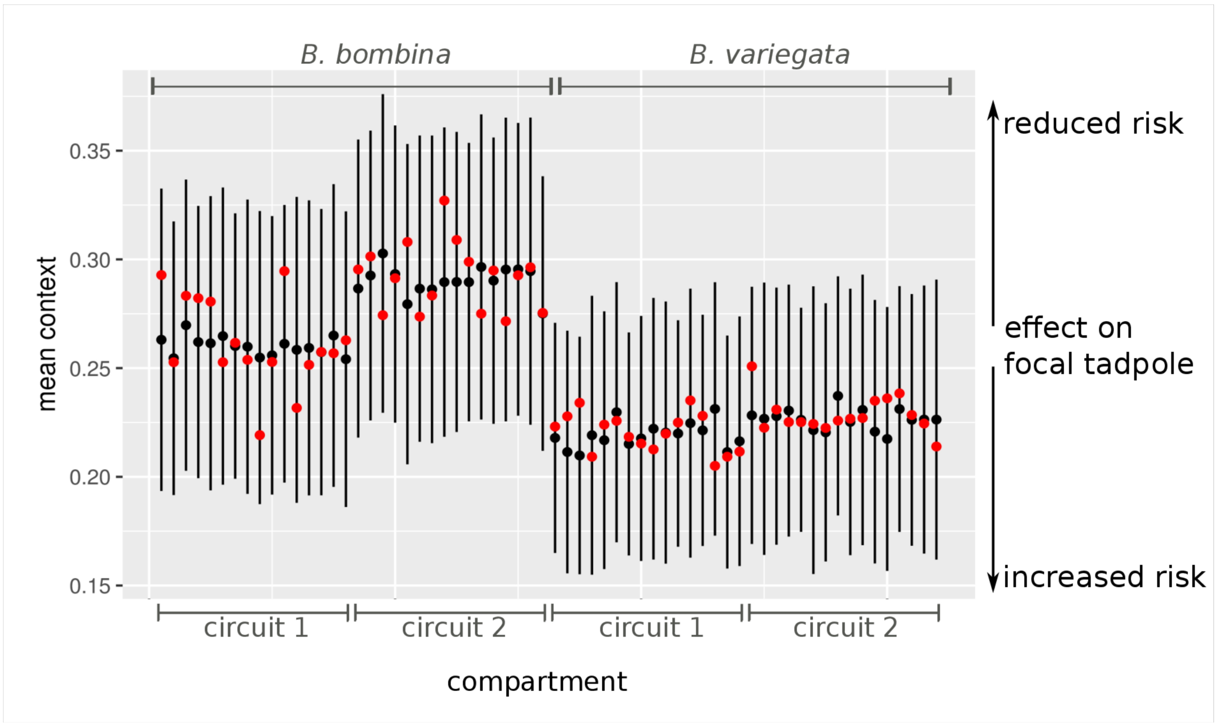

Risk contexts for the large set of hypothetical trials are shows with black dots and error bars (= 1 standard deviation). Red dots indicate observed risk contexts. Reduced/increased risk on the right hand side refers to the level of risk faced by a tadpole from the focal compartment. In the company of well defended tadpoles, the focal tadpole is at relatively higher risk and *vice versa*. For this reason, *B. bombina* are placed on average into a higher risk context than *B. variegata.*
