# Supplementary material for: Tadpoles of hybridising fire-bellied toads (B. bombina and B. variegata) differ in their susceptibility to predation
Source: PLoS One. 2020 Dec 7;15(12):e0231804. doi: 10.1371/journal.pone.0231804 (PMC7721483; doi:10.1371/journal.pone.0231804)
Supplement: S2 Fig — (PDF) [file pone.0231804.s002.pdf]

S2 Fig. Pairwise plots of morphological traits.

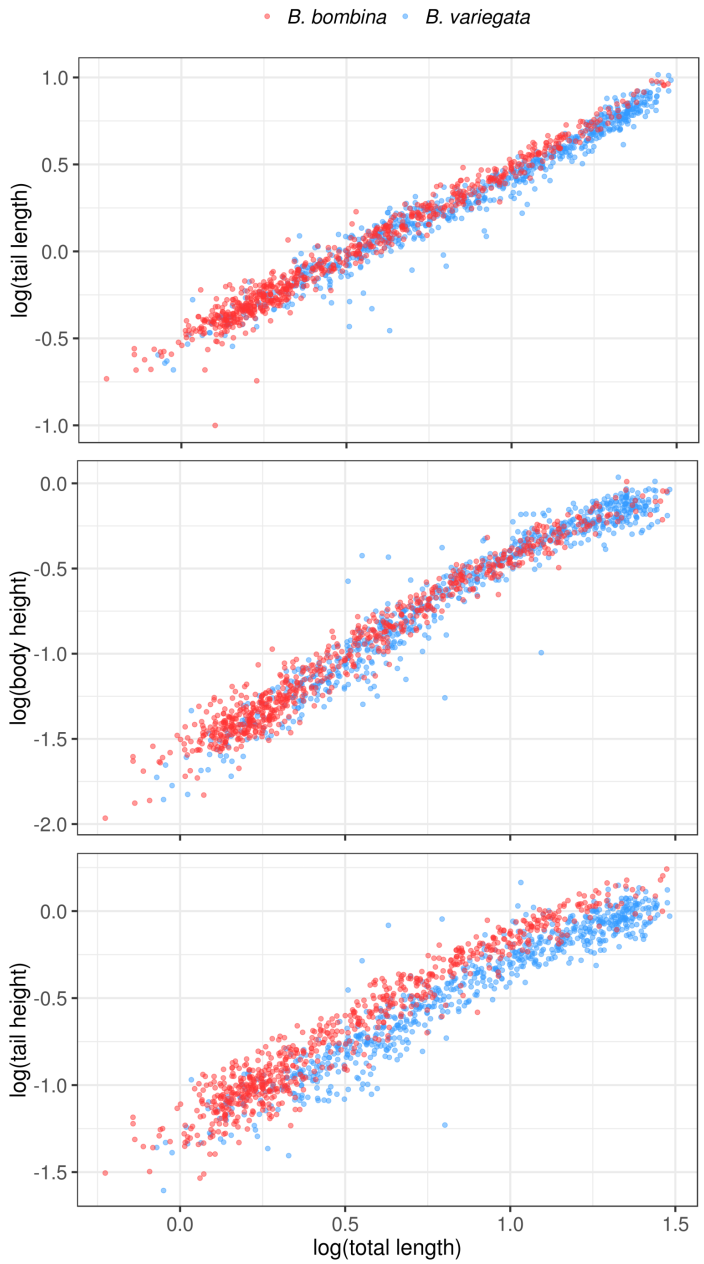

Relationships between the four morphological measurements. Parallel slopes indicate shared allometry across taxa. Note that log(tail height) shows the strongest taxon difference.
