# Supplementary material for: Tadpoles of hybridising fire-bellied toads (B. bombina and B. variegata) differ in their susceptibility to predation
Source: PLoS One. 2020 Dec 7;15(12):e0231804. doi: 10.1371/journal.pone.0231804 (PMC7721483; doi:10.1371/journal.pone.0231804)
Supplement: S1 Table — (PDF) [file pone.0231804.s003.pdf]

S1 Table. Ecological features of collection sites. Vegetation is expressed as percent cover of the water surface (aquatic vegetation: submerged, emergent), as percent of the site circumference (bank: herbacious vegetation) and in terms of the overall setting (forest: yes/no). Plant genera in or around *B. bombina* sites: mostly *Typha, Phalaris, Equisetum, Acorus, Potamogeton* and *Ceratophyllum*.

Taxon	Site	Length (m)	Width (m)	Depth (cm)	Vegetation %			Forest	Predators	Substrate
					submerged	emergent	bank			
B.v.	1A	10	1.3	20	0	0	0	yes	*Aeshna cyanea, Natrix natrix*	leaf litter
B.v.	1B	2.5	0.7	27	0	0	0	yes	*Aeshna cyanea, Natrix natrix*	leaf litter
B.v.	2	75	27	200	0	0	0	no	water bug (Nepidae)	leaf litter
B.v.	5	5.2	0.4	8	0	0	0	yes	-	mud, leaf litter
B.v.	6	3.7	0.5	12	0	0	80	yes	-	mud, leaf litter
B.v.	16	3.3	0.5	6	0	0	0	yes	*Aeshna cyanea, Natrix natrix*	mud, leaf litter
B.v.	17	1.7	0.5	15	0	0	100	no	*Aeshna cyanea*	mud, leaf litter
B.v.	18	7.5	4	21	0	0	0	yes	*Aeshna cyanea, Natrix natrix*	mud, leaf litter
B.v.	19	9.4	1	21	0	0	5	yes	*Aeshna cyanea, Natrix natrix*	mud, leaf litter
B.v.	20	3	0.65	22	0	0	100	yes	-	mud, leaf litter
B.b.	E	10	5	120	0	0	0	yes	*Aeshna cyanea, Natrix natrix*, fish	
B.b.	H	552	298	300	95	70	100	yes	*Aeshna cyanea, Natrix natrix*, fish	
B.b.	K	324	347	200	100	1	100	no	*Aeshna cyanea, Natrix natrix*, fish	
B.b.	M	173	120	300	90	80	100	no	*Aeshna cyanea, Natrix natrix*, fish	
